# Super-resolution ultrasound radiomics for pre-FNA prediction of nondiagnostic (Bethesda I) thyroid nodules

**DOI:** 10.3389/fendo.2026.1710097

**Published:** 2026-05-01

**Authors:** Shaozheng He, Guo-Rong Lyu, Mingli Cai, Jian Lin, Kunzhang Zeng, Junfa Sheng

**Affiliations:** 1 Department of Ultrasound, Second Affiliated Hospital of Fujian Medical University, Quanzhou, Fujian, China; 2 Department of Ultrasound, Jinjiang Municipal Hospital, Quanzhou, Fujian, China

**Keywords:** Bethesda I category, fine-needle aspiration (FNA), radiomics, super-resolution ultrasound, thyroid nodule

## Abstract

**Introduction:**

We evaluated whether a generative adversarial network (GAN)-based super-resolution (SR) radiomics pipeline could improve the pre-fine-needle aspiration (FNA) prediction of nondiagnostic (Bethesda I) outcomes in thyroid nodules compared with normal-resolution (NR) ultrasound analysis.

**Methods:**

This single-center retrospective study included 437 patients with thyroid nodules, with 338 assigned to the development cohort and 99 to a temporally independent validation cohort. Two-dimensional B-mode ultrasound images were processed using a pretrained GAN-based SR model in inference-only mode. Handcrafted radiomic features were extracted using PyRadiomics and subjected to a multistep selection pipeline comprising the Mann-Whitney U test, Spearman correlation-based redundancy pruning, random forest-based recursive feature elimination (RFE), and least absolute shrinkage and selection operator (LASSO) regression. Four machine-learning classifiers, including random forest (RF), LightGBM, ExtraTrees, and XGBoost, were trained using five repeated stratified 80/20 splits. Post hoc probability calibration was performed using Platt (sigmoid) scaling fitted on the training set and applied unchanged to the independent validation set. In addition, qualitative error analysis was conducted to characterize false-positive and false-negative cases and to assess the potential effect of operator experience on model performance.

**Results:**

The SR-based models consistently outperformed the NR-based models. The SR-RF model achieved an area under the receiver operating characteristic curve (AUC) of 0.808 in training and 0.733 in internal testing, compared with 0.672 and 0.596, respectively, for the NR-RF model. In the independent validation cohort, the SR-RF model yielded an AUC of 0.7435. Platt calibration improved the Brier score from 0.1885 to 0.1702 without affecting AUC, indicating improved probability reliability. Across classifiers, RF and LightGBM showed the most balanced overall performance. Qualitative error analysis showed that false-positive cases were often associated with cystic or heterogeneous echotexture and posterior acoustic artifacts, whereas false-negative cases involved isoechoic solid nodules with subtle margins and limited region-of-interest (ROI) coverage. No systematic effect of operator experience on model performance was observed.

**Discussion:**

GAN-based SR radiomics significantly improves the pre-FNA prediction of nondiagnostic cytology in thyroid nodules. When combined with post hoc calibration, this approach provides more reliable individualized risk estimates and may help reduce unnecessary repeat procedures.

## Introduction

Thyroid nodules are prevalent in the general population, and their primary clinical significance lies in excluding malignancy, which is present in approximately 5–15% of cases (1). The screening and diagnosis of thyroid nodules are central components of clinical endocrinology. With advances in ultrasound technology, fine-needle aspiration (FNA) has become the standard method for evaluating thyroid nodules (2). The 2017 Bethesda System for Reporting Thyroid Cytopathology (TBSRTC) classifies thyroid fine-needle aspiration cytology (FNAC) samples into six diagnostic categories, ranging from benign (II) to malignant (VI). Categories III (atypia/follicular lesion of undetermined significance) and IV (follicular neoplasm or suspicious for follicular neoplasm) are classified as indeterminate, carrying malignancy risks of 10–30% and 25–40%, respectively (3). However, up to 15% of FNA results are assigned to Bethesda category I, deemed “nondiagnostic” because of inadequate specimen cellularity or suboptimal sample quality. Among surgically resected Bethesda I nodules, reported malignancy rates range from 9% to 32% (4). Bethesda category I nodules often necessitate repeat aspiration or further diagnostic workup, which increases both the psychological and physical burden on patients and contributes to additional healthcare costs. Furthermore, transient cytologic atypia in follicular cells induced by prior FNA may confound subsequent cytologic interpretation (3). In addition, certain cases remain indeterminate even after multiple repeat aspirations of the same nodule (5). Predicting whether a nodule will produce a Bethesda I result before the initial aspiration remains a major challenge in clinical decision-making.

In a recent study, Jintao Wu et al. (6) explored the diagnostic significance of subjective puncture sensations—specifically, the stiffness and grittiness perceived by the operator during FNA—in differentiating benign from malignant nodules with indeterminate cytology. The presence of at least one puncture sensation achieved a sensitivity of 79.2% and a specificity of 75.0%, indicating moderate diagnostic performance. However, the principal limitation of this approach is its inherent subjectivity: all FNAs were performed by a single interventional sonographer with more than 10 years of experience, and the puncture sensations were verbally reported without standardized criteria. This heavy reliance on operator expertise compromises reproducibility, particularly in routine clinical environments or low-resource settings. Moreover, because puncture sensation is an intraoperative phenomenon, it cannot be utilized for pre-FNA decision-making or early patient stratification.

As an emerging technology, ultrasound radiomics provides a comprehensive and objective assessment of thyroid nodules by extracting a wide range of quantitative features from ultrasound images. By analyzing medical images at a fine-grained level, radiomics facilitates comprehensive tumor characterization and promotes a transition from subjective visual interpretation to large-scale, data-driven analysis (7). Radiomics has demonstrated broad utility across oncology (8, 9). These developments open new possibilities for predicting the occurrence of Bethesda category I nodules. Despite these advances, the predictive capability of radiomics remains constrained by the quality of input images. Normal-resolution (NR) ultrasound images often fail to delineate the margins of thyroid nodules clearly and hinder visualization of internal structures, particularly in smaller lesions. This limitation substantially reduces the accuracy of risk stratification and impairs diagnostic and therapeutic precision. Therefore, enhancing image resolution is crucial for achieving accurate risk stratification and reliable diagnosis.

Edge blurring and block-like artifacts are common limitations of interpolation-based resolution enhancement. Deep learning–based super-resolution (SR) techniques have recently emerged as data-driven approaches to enhance spatial detail in medical images without hardware modification (10–14). In this study, SR was implemented using a generative adversarial network (GAN) architecture to improve spatial fidelity and fine textural representation in conventional B-mode ultrasound images. By reconstructing plausible high-frequency details and reducing interpolation artifacts, SR may provide a richer foundation for radiomic feature extraction and subsequent machine-learning analysis.

This study aims to evaluate the potential of SR ultrasound radiomics in predicting whether the FNA result of a thyroid nodule will be classified as Bethesda category I. By analyzing diverse radiomic features extracted from ultrasound images, this study seeks to develop a novel decision-support tool to assist clinicians in reducing repeat aspirations and enhancing diagnostic accuracy.

## Methods

### Patient population, ethics approval, and definitions

This study was approved by the Ethics Committee of Jinjiang Municipal Hospital (approval No. jjsyyll-2025-166), and the requirement for informed consent was waived due to its retrospective design. Patients who underwent thyroid FNA at Jinjiang Municipal Hospital between April 2024 and April 2025 were retrospectively enrolled for model development. An independent temporal validation cohort, consisting of patients who underwent FNA between May and August 2025, was collected separately to assess the generalizability of the developed models. All cases were retrieved from the electronic medical record systems of the Departments of Ultrasound and Pathology. Only nodules classified as C-TI-RADS category 4A or higher with a clear clinical indication for FNA were included in this study. Based on this predefined inclusion strategy, the study cohort primarily consisted of solid or predominantly solid nodules, reflecting real-world clinical practice under C-TI-RADS–guided management.

The inclusion and exclusion criteria were defined as follows. Inclusion criteria: (1) patients who underwent conventional gray-scale ultrasound with diagnostically adequate image quality; (2) a definite cytology result from the first FNA, classified as any Bethesda category (I–VI) according to the 2017 TBSRTC; and (3) nodules with well-defined margins and a clearly identifiable region of interest (ROI). Exclusion criteria: (1) poor image quality or severe imaging artifacts; (2) multiple nodules in which the aspirated site could not be accurately matched to the corresponding ultrasound image; and (3) incomplete clinical or pathological data.

Outcome definition and grouping: The supervised prediction target was whether the initial FNA would result in a non-diagnostic cytology (Bethesda I). For analytical purposes, outcomes were binarized as “Bethesda I” (positive class) versus “non-I” (negative class; Bethesda II–VI). All Bethesda categories (I–VI) were included to represent the full spectrum of real-world FNAC outcomes and to construct a clinically meaningful negative class comprising diagnostic categories (II–VI). Although Bethesda categories II–VI differ in malignancy risk and subsequent management, they all provide actionable cytological information, in contrast to Bethesda I, which typically necessitates repeat procedures, diagnostic delays, and additional costs. To prevent information leakage, only the first FNAC result per nodule or patient was used for labeling; subsequent aspirations were excluded from endpoint definition. Throughout the study, the two analytical groups were defined as “Bethesda I” and “non-I, ” with Bethesda I designated as the positive class for all discrimination, calibration, and decision-curve analyses.

### Image acquisition and processing

All ultrasound examinations were conducted following a standardized protocol to ensure consistency and reproducibility in radiomic feature extraction. Conventional B-mode ultrasound imaging was performed on a GE LOGIQ E9 scanner equipped with a high-frequency linear array transducer (4.5–12.0 MHz). All imaging was acquired with patients in the supine position, and operators were blinded to the final pathological outcomes. Maximal transverse two-dimensional gray-scale images of the nodules were obtained before FNA.

**Figure 1:**
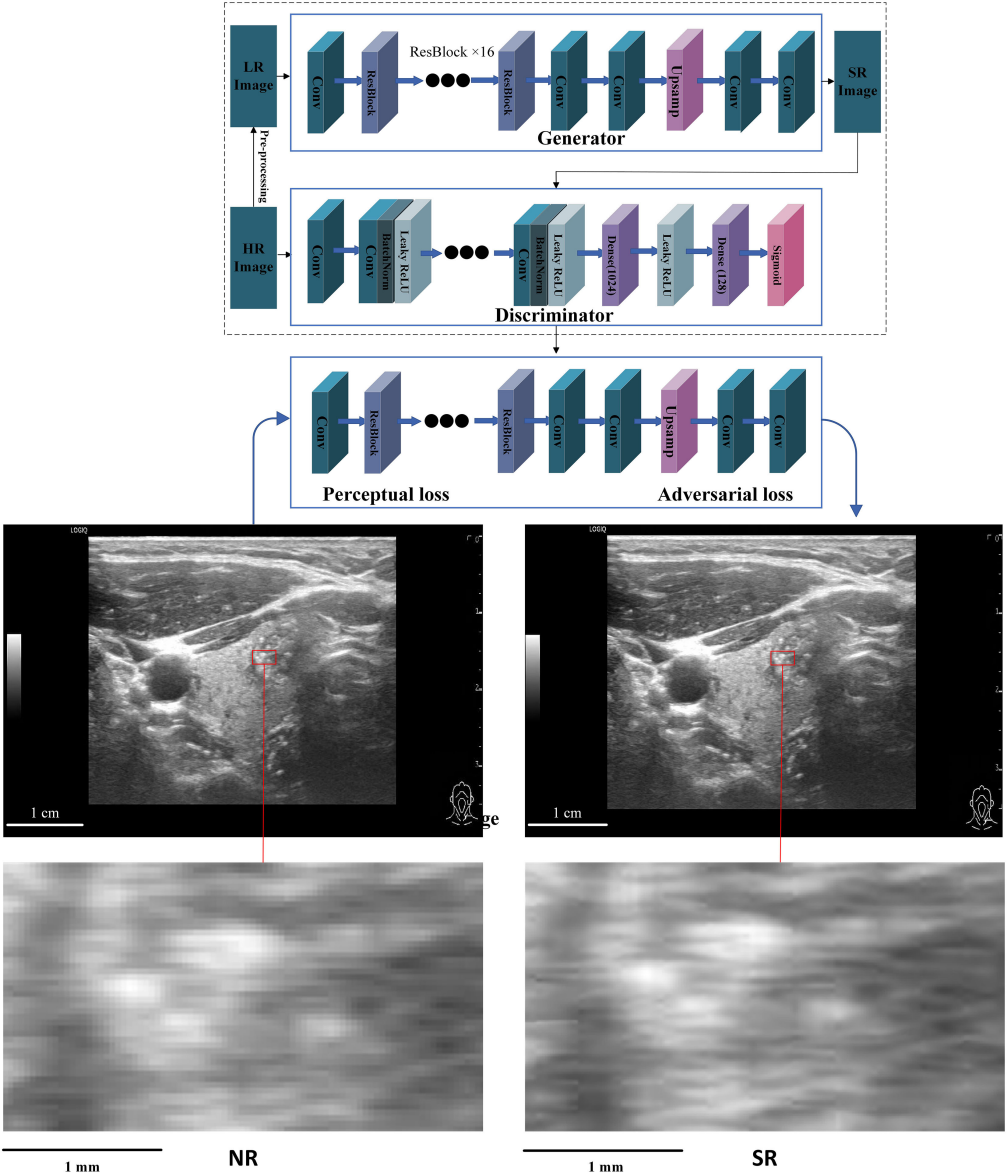
GAN-based super-resolution pipeline and NR–SR image comparison.Top: GAN-based SR pipeline. Middle: NR (left) and SR (right) images of the same nodule. Red boxes indicate regions magnified below for visual comparison (not ROIs). SR denotes GAN-based computational upscaling/detail enhancement of B-mode ultrasound. Scale bars: 1 cm (original), 1 mm (magnified). NR, normal-resolution; SR, super-resolution; GAN, generative adversarial network.

SR reconstruction was performed with a vendor-provided, pretrained GAN-based model on the OnekeyAI platform (v4.10.27; OnekeyAlgo framework). The implementation follows the Enhanced Super-Resolution Generative Adversarial Network (ESRGAN) architecture, using a residual-in-residual dense block (RRDB) generator and a PatchGAN-style convolutional discriminator (**Figure 1**) (15, 16). The model was used for inference only, with no additional training or fine-tuning.

Input ultrasound images were first converted to the*.nii.gz* format. High-resolution SR reconstructions were then generated during SR inference using OnekeyAlgo with a 4× scaling factor (scale = 4) under default settings (pretrained weights, network configuration, and fixed hyperparameters). Accordingly, training-related parameters (e.g., learning rate, batch size, number of epochs, and data augmentation) were not modified and the model was not retrained in this study.

The SR model employed a composite loss function integrating pixel-wise L1 loss, VGG-basedperceptual loss, and adversarial loss to preserve both global structure and local textural fidelity.A summary of the SR model’s inference configuration is presented in Supplementary **Table S1** (10, 17). The SR pipeline used in this study is described in the **Supplementary Materials**, which include a demonstration Jupyter notebook, configuration files, executable scripts, and environment specifications (see **Supplementary Files S1**–**S4**; **Supplementary Table S1**). These materials illustrate the workflow and parameter settings for transparency and academic reproducibility. The provided package contains illustrative or compiled components intended for methodological demonstration and does not represent an open-source software release.

### Operator experience variables

To account for potential variability in procedural outcomes arising from operator-dependent technical factors, quantitative measures of operator experience were incorporated into the analysis. For each operator, the following variables were recorded and calculated:

Years of experience: Defined as the number of full years since the operator began independently performing FNA procedures and treated as a continuous variable.

Operator case count (within-study): Defined as the number of FNA procedures performed by each operator among the 338 patients included in the study, used to reflect procedural volume under consistent imaging and reporting standards.

Experience-weighted exposure index (EWI): A composite variable integrating both operator seniority and procedural volume, defined as follows:

EWI=Years of experience×Number of FNA cases within the study

A multiplicative integration was chosen to emphasize the joint contribution of clinical seniority and procedural volume to diagnostic performance. This multiplicative approach amplifies the influence of operators with extensive experience and high procedural volume, providing a more accurate representation of cumulative procedural exposure than additive or averaged indices.

Given the small number of operators (n = 7), we did not perform cluster-robust regression inference. All analyses were descriptive and exploratory, with group comparisons conducted using the unadjusted Mann–Whitney U test.

### Image segmentation and extraction of radiomic features

Analyses were conducted on two-dimensional (2D) ultrasound images. To ensure reproducibility, images underwent preprocessing before feature extraction. Both SR and NR images were resampled to a uniform in-plane spacing of [1.0, 1.0] (2D) using B-spline interpolation (sitkBSpline) prior to PyRadiomics feature extraction. Within the ROI, grayscale intensities were z-score normalized (normalize: true in PyRadiomics), and a 3×3 median filter was applied for mild denoising (18). ROIs were manually delineated in ITK-SNAP (v3.8) by two sonographers (5 and 10 years of experience in thyroid ultrasound), blinded to cytology results. ROIs were restricted to the lesion itself (excluding perilesional tissue), and disagreements were resolved by consensus. From the original images, 107 features were extracted per lesion (18 first-order, 75 textures, 14 shape), with gray-level discretization set to a bin width of 5. The parameter file used for feature extraction (**Supplementary File S5**, exampleUS.yaml) and the complete list of 107 features (**Supplementary Table S2**) are provided in the **Supplementary Materials**.

### Feature selection and regularization strategy

To ensure robustness and interpretability of radiomic modeling, a multi-step feature selection pipeline was adopted.

Intra- and inter-observer reproducibility screening: Only features with an intraclass correlation coefficient (ICC) ≥ 0.75 for both intra- and inter-observer assessments were retained, ensuring that only stable features across repeated ROI delineations were included. Reproducibility screening was conducted separately in the SR and NR domains using intra- and inter-observer ICC analysis.

Because NR images were derived from identical ROIs and underwent the same preprocessing and discretization procedures, segmentation-related reproducibility is expected to be comparable across domains. Domain-specific ICC analyses were not separately repeated for NR images, which should be addressed in future methodological studies.

Univariate filtering (Mann–Whitney U test): A non-parametric Mann–Whitney U test was used to compare feature distributions between Bethesda I and non-I nodules in the training set. Features with p ≥ 0.05 were excluded from subsequent analysis to remove weakly discriminative variables.

Redundancy pruning (Spearman correlation): Pairwise Spearman correlation coefficients were calculated among the retained features. For any pair with |ρ| > 0.90, the feature with the higher p-value from the Mann–Whitney test was discarded to minimize multicollinearity and dimensional redundancy.

Model-based preselection (recursive feature elimination, RFE): Random Forest (RF)–based RFE was used to select a compact subset of features before least absolute shrinkage and selection operator (LASSO). A fivefold stratified cross-validation was applied within each training fold, with a step size of 1 and a minimum feature count of 20. This step helped prevent overfitting by eliminating weakly informative features.

Final selection (LASSO logistic regression): LASSO logistic regression was applied to the RFE-preselected features. Stratified tenfold cross-validation was used to select the regularization parameter λ based on the minimum mean squared error criterion. Features with nonzero coefficients at the optimal λ (λ_opt) were retained as the final radiomic signature.

Hyperparameters were prespecified based on prior methodological experience and small-sample considerations rather than optimized through data-driven search. To assess selection stability, the retention frequency of final LASSO-selected features was quantified across five independent resampling iterations. The final selected features and λ values are reported in the Results section.

### Model development, class imbalance handling, and evaluation strategy

Model development employed a repeated random-subsampling scheme. Across five runs with distinct random seeds, the dataset was split 80:20 into training and test sets. Within each run, all feature selection, model fitting, and internal evaluation were conducted exclusively on the training set to prevent information leakage. Stratified splits were used to preserve the empirical class proportions between Bethesda I and Bethesda II–VI nodules.

In the primary analysis, no over-sampling or under-sampling was applied so that model performance reflected the natural class imbalance. An optional Synthetic Minority Oversampling Technique (SMOTE) step was implemented after feature selection and before model training; SMOTE was applied only to training folds to prevent data leakage. This step was controlled by a pipeline toggle (use_smote) and was disabled by default.

Four tree-based classifiers were evaluated: RF, XGBoost, LightGBM, and ExtraTrees. Hyperparameters were manually prespecified and held constant across outer runs (see **Supplementary Table S3**). Rather than performing exhaustive grid search optimization, conservative model configurations were defined *a priori* to reduce the risk of data-driven overfitting and to ensure consistent comparison between SR- and NR-based pipelines. Given the modest number of Bethesda I events and the low effective dimensionality after feature selection, model capacity was intentionally constrained. For tree-based models, ensemble size and tree depth were selected within a conservative structural range appropriate for small-sample classification settings, balancing nonlinear modeling capacity with variance control. More complex configurations were not pursued in order to prioritize structural stability, reproducibility, and generalizability. Decision thresholds for threshold-dependent metrics were determined using Youden’s J statistic on the training set and applied unchanged to the corresponding test set.

For visualizations (ROC, calibration, and decision-curve analysis [DCA]), the iteration whose area under the receiver-operating-characteristic curve (AUC) was closest to the five-run median AUC was selected to avoid over- or under-representing performance. Calibration curves were plotted for both training and test sets before and after Platt scaling. Decision-curve analysis was performed across threshold probabilities from 0 to 1, with thresholds of 11%, 16%, and 21% highlighted as clinically meaningful. Final results were averaged across the five runs and reported as mean ± SD, with 95% confidence intervals (CIs) provided for each metric.

### Baseline model using conventional ultrasound (C-TI-RADS-type) features

In addition to the radiomics classifiers above, we constructed a conventional ultrasound baseline logistic regression using routinely reported C-TI-RADS descriptors (solid composition, marked hypoechogenicity, taller-than-wide, microcalcification/punctate echogenic foci, and irregular margin/extrathyroidal extension) together with nodule size. Nodule size was defined as the longest diameter measured on the maximal cross-sectional plane (Dmax, mm), computed from two orthogonal diameters (Dmax = max (D1, D2)). The baseline model was evaluated under the same five independent stratified 80/20 splits and identical evaluation procedures as the SR-radiomics models, with all preprocessing fitted on the training set only and applied to the held-out test set, including within-split standardization of Dmax using training-set statistics.

### Independent validation and calibration analysis

To assess and improve probability calibration, we used *post-hoc* Platt scaling (sigmoid/logistic regression) as the primary calibration method. The workflow was as follows: (i) all predicted probabilities were oriented to the positive class P (y = 1); (ii) a logistic-regression calibrator was fitted only on the development (training/calibration) data with five-fold internal cross-validation to mitigate overfitting; and (iii) the fitted mapping was transported unchanged to the independent temporal validation cohort to evaluate out-of-sample calibration. Calibration was assessed using reliability diagrams with 10 equal-width bins and the Brier score; discrimination was summarized by the AUC.

### Statistical analysis

All statistical analyses were performed using SPSS (v25.0; IBM) and Python (v3.7) with scikit-learn. Model performance was evaluated across three dimensions: discrimination, calibration, and clinical utility. Continuous variables were tested for normality using the Shapiro–Wilk test. Normally distributed variables were summarized as mean ± SD and compared using independent-samples t-tests; non-normally distributed variables were reported as median (interquartile range, IQR) and compared using the Mann–Whitney U test. Categorical variables were reported as frequencies (n, %) and compared using χ² tests or Fisher’s exact test, as appropriate.

Pairwise AUCs were compared using the two-sided DeLong test for correlated ROC curves; Benjamini–Hochberg false discovery rate (FDR) controlled multiplicity within each evaluation set (training, internal test, and temporal validation) (FDR-adjusted p < 0.05 was considered statistically significant). In addition, accuracy, sensitivity, specificity, F1 score, positive predictive value (PPV), and negative predictive value (NPV) were reported to summarize classification performance. To address outcome imbalance, emphasis was placed on sensitivity, F1 score, and NPV as informative threshold-dependent metrics. Calibration was assessed using calibration plots, the Hosmer–Lemeshow (HL) goodness-of-fit test, and the Brier score. The operating threshold was defined by Youden’s J (maximizing sensitivity + specificity − 1) on the development set (training/calibration) and transported unchanged to the internal test sets and the independent temporal validation cohort. Clinical utility was evaluated using DCA across a prespecified range of threshold probabilities.

For internal experiments employing repeated subsampling (five independent stratified 80/20 splits), performance metrics were summarized as mean ± SD. The 95% CIs for the mean were calculated as mean ± 1.96 × SD/√5. For visualizations (ROC, calibration, and DCA), we displayed the iteration whose AUC was closest to the five-run median. For paired SR versus NR feature comparisons, SR and NR feature tables were aligned by patient identifier, and only features present in both extractions were analyzed. Missing values were handled on a per-feature pairwise complete-case basis; statistical tests were performed only on patient pairs with non-missing values for that feature. For each feature, summary statistics for SR and NR (mean, median, and mean difference SR–NR) were reported, together with a two-sided Wilcoxon signed-rank test for paired differences and the Spearman rank correlation coefficient (ρ) to quantify preservation of inter-subject ranking. All paired Wilcoxon p values were adjusted for multiple comparisons using the Benjamini–Hochberg FDR procedure; features with FDR-adjusted p < 0.05 were considered statistically significant. If the Wilcoxon test could not be computed for a feature (e.g., identical paired values), a two-sided sign test was used as a fallback, or the feature was reported with a note indicating no variability (19).

## Results

### Patient cohorts and study design

**Figure 2:**
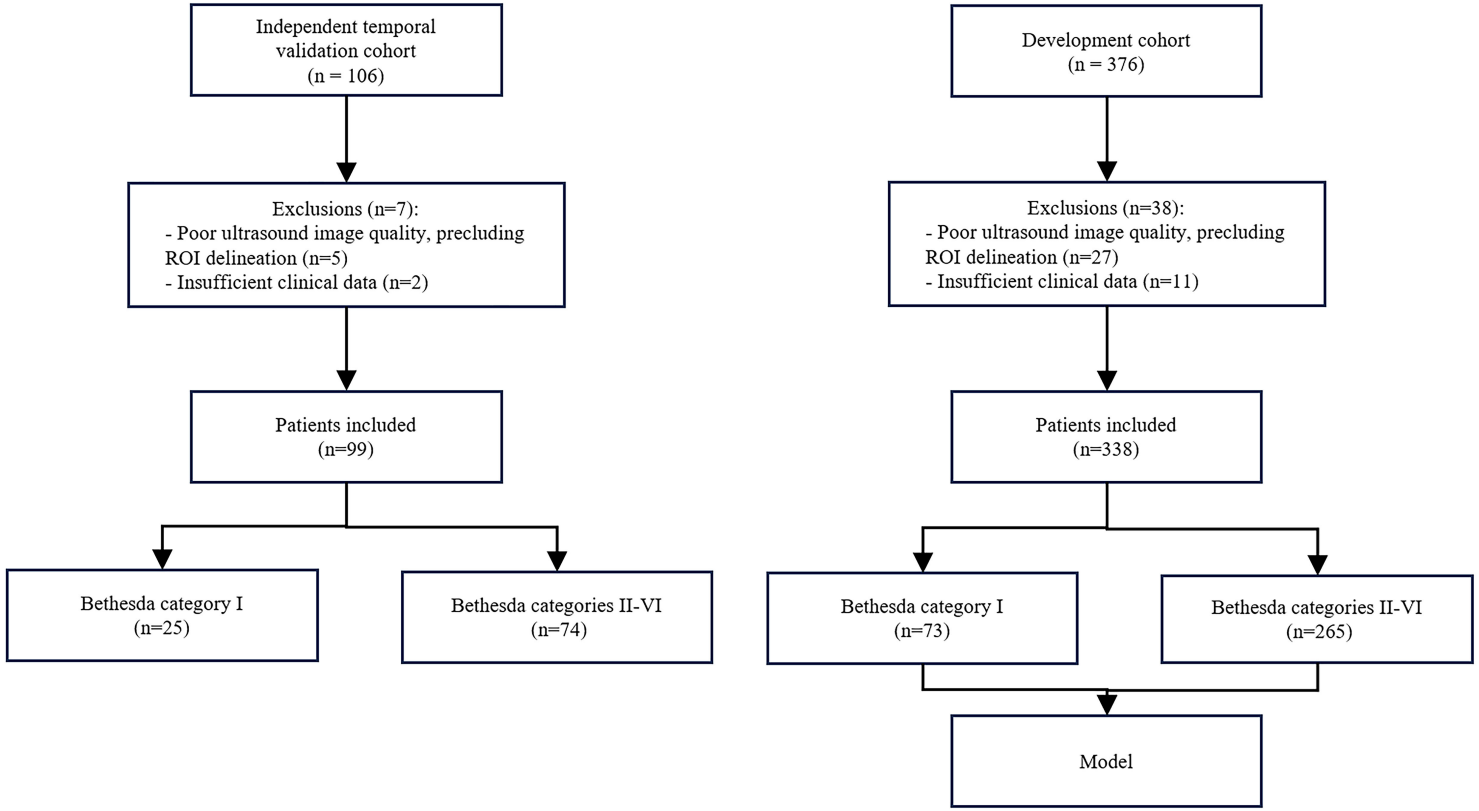
Participant inclusion and exclusion flowchart, illustrating the division of cases into the development and temporally independent validation cohorts.

From all available cytology records, 376 patients were identified. Ultimately, 338 patients met the inclusion criteria; 73 (21.6%) were classified as Bethesda category I (**Figure 2**), with a mean age of 46.9 ± 9.9 years. The remaining 265 patients were classified as Bethesda categories II–VI, with a mean age of 44.4 ± 11.0 years. The between-group age difference was not significant (independent-samples t-test: t = −1.787; p = 0.075). Sex distribution did not differ significantly between groups (Bethesda I: 15 men, 58 women; Bethesda II–VI: 75 men, 190 women; χ² (1) = 1.76, p = 0.184). Regarding nodule composition, no purely cystic nodules were included (0/338, 0%), and no nodules were predominantly cystic (defined as a cystic component >50%; 0/338, 0%). The cohort was highly enriched for solid/almost solid nodules (320/338, 94.7%), with only 18/338 (5.3%) exhibiting mixed solid–cystic composition. All procedures were performed by seven operators. An independent temporal validation cohort of 99 patients was included for out-of-time (temporal) validation. As summarized in **Table 1**, there were no significant differences in age, sex, or Bethesda category distribution between the development and validation cohorts (all p > 0.3); see **Table 1** for exact p-values and test details.

**Table 1:** Baseline characteristics of the development cohort and the independent temporal validation cohort.

Characteristic	Development cohort	Temporal validation cohort	Test statistic	P
Included, n	338	99	–	–
Bethesda I, n (%)	73 (21.6%)	25 (25.5%)	χ² (1) =0.667	P=0.414
Bethesda II-VI, n (%)	265 (78.4%)	73 (74.5%)	–	–
Age, mean ± SD (years)	44.9 ± 10.8	45.8 ± 11.3	t=−0.701	P=0.483
Female, n (%)	248 (73.4%)	67 (68.4%)	χ² (1) =0.949	P=0.330
Male, n (%)	90 (26.7%)	31 (31.6%)	–	–

### Impact of operator experience on non-diagnostic (Bethesda I) outcomes

In the development cohort (n = 338), the frequency distribution of unique EWI values was: 1 (n = 1), 11 (n = 11), 15 (n = 1), 198 (n = 66), 770 (n = 77), 1100 (n = 55), and 1524 (n = 127). Descriptive statistics by outcome were: non-I (n = 265), median EWI 1100 (IQR 770–1524), mean 988.7 (SD 503.8); I (n = 73), median EWI 1100 (IQR 198–1524), mean 884.1 (SD 608.4). The unadjusted Mann–Whitney comparison showed no significant difference between groups (Mann–Whitney U = 8864.5; p = 0.256).

### Radiomic feature reproducibility

In the SR domain, 83 features demonstrated intra-observer ICC ≥0.75 and 82 demonstrated inter-observer ICC ≥0.75, with 82 features retained after applying both criteria. In the NR domain, 85 features demonstrated intra-observer ICC ≥0.75 and 79 demonstrated inter-observer ICC ≥0.75, with 79 features meeting both thresholds. The overall reproducibility patterns were comparable between domains. Most first-order and shape features, together with the majority of Gray-level co-occurrence matrix (GLCM), gray-level run-length matrix (GLRLM) and gray-level size zone matrix (GLSZM) texture features, exhibited high reproducibility. Notably, original_glszm_LargeAreaLowGrayLevelEmphasis, original_firstorder_Median, and original_glrlm_RunVariance showed ICCs > 0.95 in both intra- and inter-observer assessments. Features with ICC < 0.75 were excluded from subsequent analysis. An ICC heatmap summarizing intra- and inter-observer reproducibility in the SR domain is presented in **Supplementary Figure S1**. These results support the stability of SR-derived radiomic features and their suitability for robust predictive modeling.

### Radiomic feature selection

A stepwise feature-selection pipeline was applied to both SR and NR radiomics to identify robust and informative features for model construction. Starting from 107 initial features (18 first-order, 75 textures, 14 shape), five filtering and reduction steps were performed: ICC, univariate analysis (Mann–Whitney U test), multicollinearity removal (Spearman correlation), RFE, and LASSO regularization. Only features with ICC ≥ 0.75 in both intra- and inter-observer assessments were retained after the ICC stage.

For SR radiomics, 82 features passed ICC screening; 64 remained after Mann–Whitney U testing; 41 persisted after Spearman redundancy pruning; RFE reduced the set to 22; and a 10-fold cross-validated LASSO on the development set yielded a final signature with seven non-zero-coefficient features (final locked λ ≈ 0.0110). For NR radiomics, 79 features passed ICC screening; 68 remained after Mann–Whitney U testing; 39 after Spearman pruning; RFE selected 20; and LASSO yielded a six-feature signature (final locked λ ≈ 0.0095). Stage-wise retention counts are summarized in **Supplementary Table S4**. The complete LASSO signatures (feature names, families, coefficients) and the final locked λ for each model are provided in **Supplementary Table S5**.

Paired SR–NR comparisons demonstrated systematic, feature-class–specific effects of super-resolution on radiomic quantification. In the final LASSO-derived signatures, the SR model retained seven features and the NR model retained six (two features were shared), yielding eleven unique features in total (full lists and LASSO coefficients are given in **Supplementary Table S5**). Of these eleven features, ten exhibited significant SR–NR differences after Benjamini–Hochberg FDR correction (10/11; FDR-adjusted p < 0.05). Although many texture metrics shifted in absolute value following SR reconstruction, relative inter-subject ordering was largely preserved: the median Spearman correlation coefficient across the eleven features was 0.8727 (mean Spearman ρ = 0.8479), and all eleven features showed Spearman ρ ≥ 0.5. Summary statistics for each feature—including n_pairs, mean_SR, mean_NR, mean difference, Wilcoxon statistic and p, FDR-adjusted p, and Spearman ρ—are provided in **Supplementary Table S6**, with corresponding Bland–Altman plots and paired comparisons visualized in **Supplementary Figure S2**.

### Diagnostic performance: NR versus SR image models

**Figure 3:**
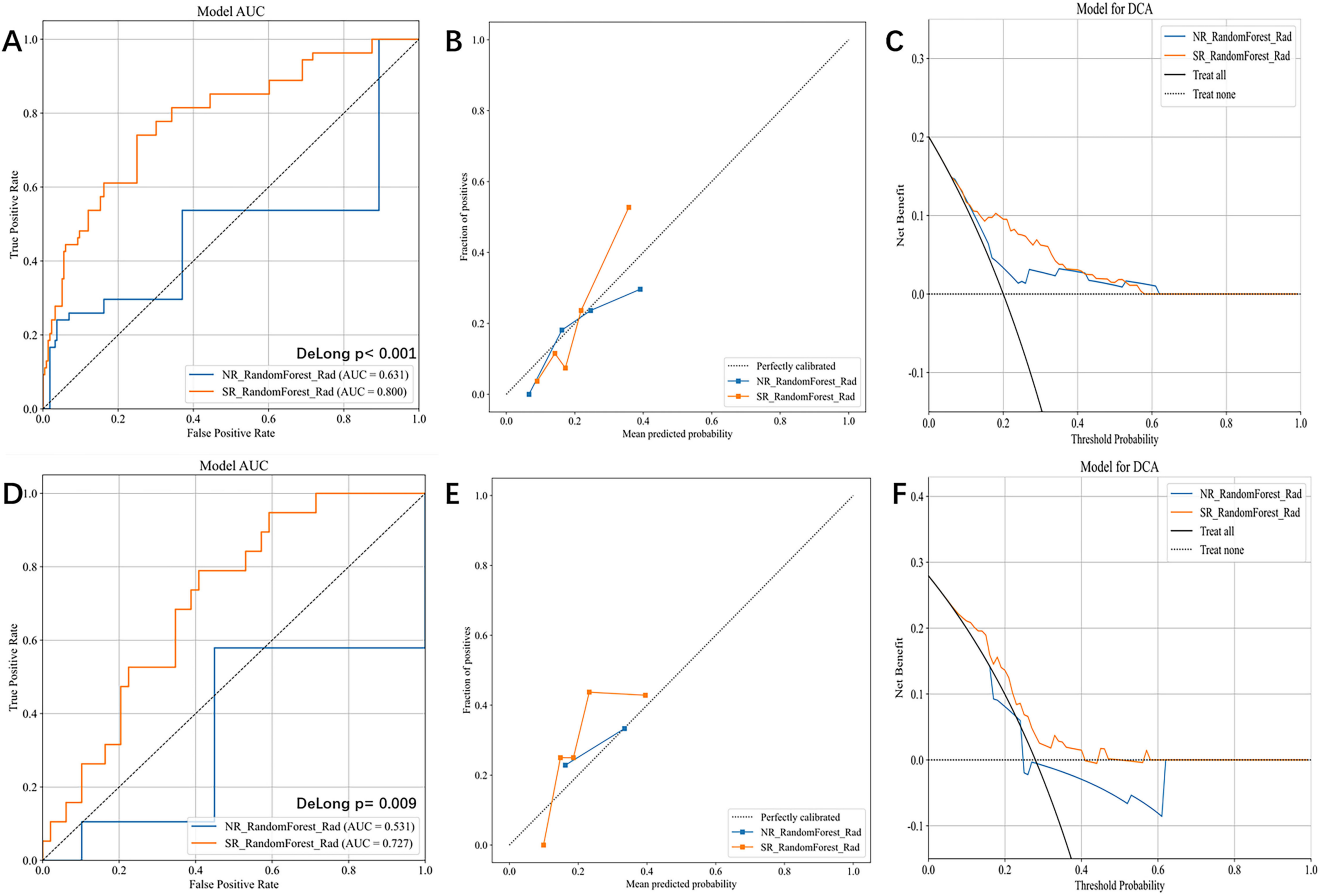
Performance comparison of SR-RF and NR-RF models.Panels **(A–C)** correspond to the training set, and panels **(D–F)** to the test set. **(A, D)** ROC curves with area under the curve (AUC) reported in the legends; p-values from the DeLong test are printed on the plots. **(B, E)** Calibration (reliability) plots: points show observed event rates versus mean predicted probabilities within bins; the dashed diagonal denotes perfect calibration. **(C, F)** Decision-curve analysis (DCA): assessment of the clinical utility of using the model across threshold probabilities from 0 to 1, relative to the “Treat all” and “Treat none” reference strategies. In all panels, blue = NR_RandomForest_Rad and orange = SR_RandomForest_Rad. SR, super-resolution; NR, normal-resolution; RF, random forest; ROC, receiver-operating characteristic; AUC, area under the curve; DCA, decision-curve analysis.

In the training sets, the NR model yielded an AUC of 0.672 ± 0.039, and in the test sets an AUC of 0.596 ± 0.055. By contrast, the SR model demonstrated superior discrimination, achieving AUCs of 0.808 ± 0.017 (training) and 0.733 ± 0.072 (test). **Table 2**, **Figure 3** (Panels A–C: training set; Panels D–F: test set) summarize the complete performance profiles for both models, including accuracy, sensitivity, specificity, PPV, NPV, F1 score, AUC, and HL calibration statistics (Comprehensive 95% CIs for all metrics of the RF model are reported in **Supplementary Table S7**).

**Table 2:** Performance comparison between NR and SR image models.

Model	Accuracy	AUC	AUC 95% CI	Sensitivity	Specificity	PPV	NPV	F1	Hosmer- Lemeshow test(p)
NR train	0.721 ± 0.021	0.672 ± 0.039	0.638 - 0.706	0.454 ± 0.118	0.793 ± 0.047	0.373 ± 0.038	0.843 ± 0.019	0.359 ± 0.117	0.985
SR train	0.729 ± 0.049	0.808 ± 0.017	0.793 - 0.823	0.735 ± 0.083	0.728 ± 0.079	0.436 ± 0.046	0.909 ± 0.017	0.543 ± 0.034	0.064
NR test	0.609 ± 0.124	0.596 ± 0.055	0.548 - 0.644	0.510 ± 0.137	0.645 ± 0.126	0.285 ± 0.045	0.843 ± 0.076	0.312 ± 0.144	0.703
SR test	0.680 ± 0.086	0.733 ± 0.072	0.670 - 0.796	0.703 ± 0.078	0.676 ± 0.120	0.384 ± 0.129	0.896 ± 0.033	0.479 ± 0.088	0.369

Additionally, compared with the C-TI-RADS-type baseline logistic regression (mean test AUC 0.584, 95% CI 0.538–0.630), the best-performing SR-radiomics model achieved a higher mean test AUC of 0.733 (95% CI 0.670–0.796; ΔAUC = 0.149), and this improvement was statistically significant (DeLong test, p <0.05).

In the development cohort, the SR-based RF model demonstrated variability across five repeated stratified splits, with a mean test AUC of 0.728 ± 0.062. The development dataset included 73 Bethesda I events and 7 final model variables, yielding an events-per-variable (EPV) ratio of 10.4, which exceeds the commonly cited heuristic threshold of 10 for regression-based modeling. Across these five splits, three of the seven radiomic features were retained in all folds (selection rate = 100%), whereas the remaining four were retained in ≥40% of folds (**Supplementary Table S8**). Coefficient directions remained consistent across splits with small standard deviations, supporting overall feature stability. Decision thresholds determined using Youden’s J statistic on the training folds ranged from 0.182 to 0.266 across five repetitions (mean ± SD: 0.220 ± 0.032). These thresholds were subsequently applied unchanged to the corresponding internal test sets and the temporal validation cohort.

### Comparison of predictive performance across multiple classifiers

**Figure 4:**
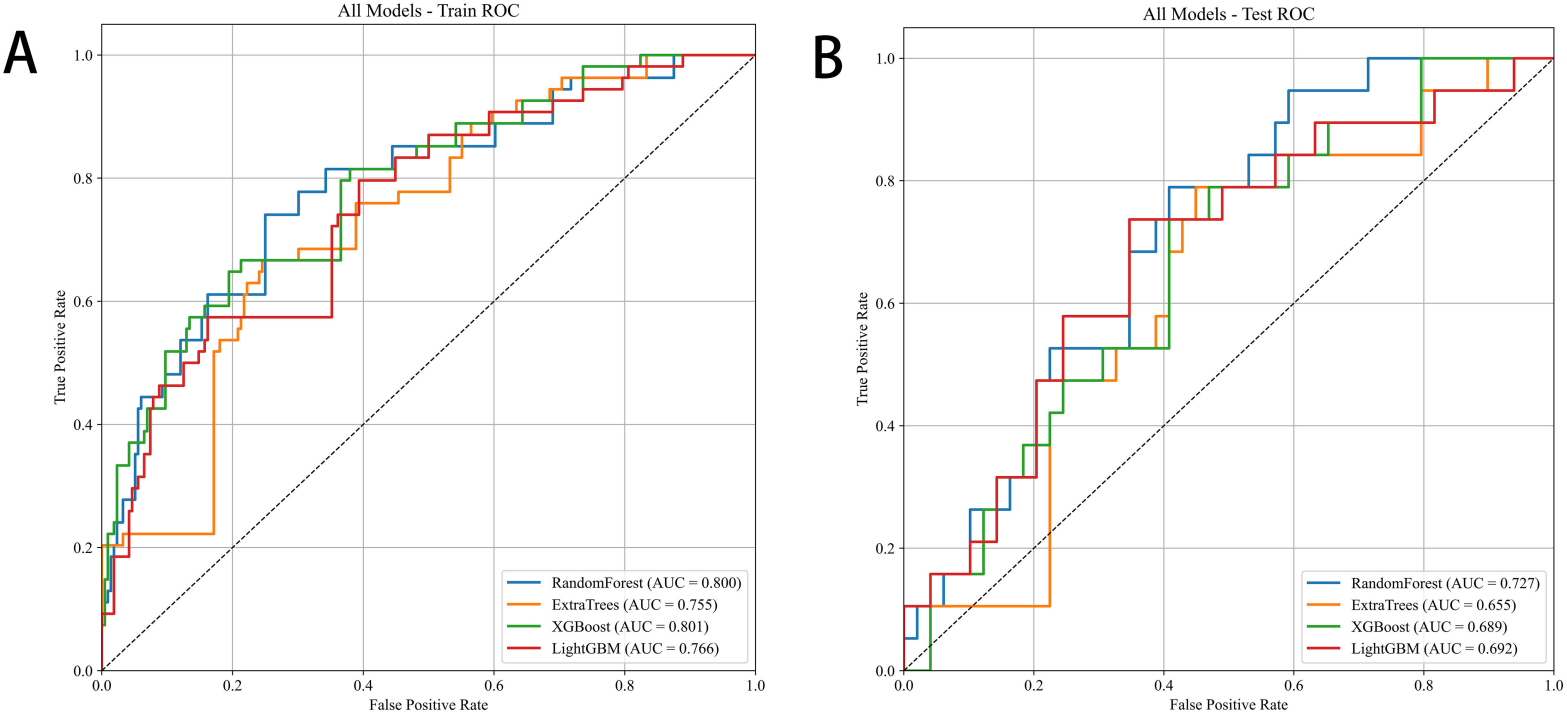
ROC curve comparison of four machine learning classifiers. **(A)**: Training set; **(B)**: Test set.

To evaluate and compare the predictive capabilities of different machine-learning classifiers, we performed repeated random subsampling (five iterations) on the training and test datasets derived from SR and NR ultrasound images. The evaluated models included RF, ExtraTrees, XGBoost, and LightGBM (**Figure 4**; **Supplementary Table S7**).

Across NR and SR datasets, predictive performance ranged from moderate to high; by AUC and overall accuracy, RF and LightGBM consistently ranked in the top tier. In the SR dataset, RF achieved the highest training AUC (0.808 ± 0.017) and a robust test AUC (0.733 ± 0.072), indicating good generalization. LightGBM showed similar stability (training AUC 0.773 ± 0.014; test AUC 0.732 ± 0.098).

For the NR dataset, performance was generally lower. The highest training AUC was observed for LightGBM (0.676 ± 0.044), while test AUCs for all models remained below 0.60. ExtraTrees and XGBoost produced relatively low sensitivity and F1 scores on the test set, suggesting a trade-off between specificity and recall for those classifiers.

**Figure 5:**
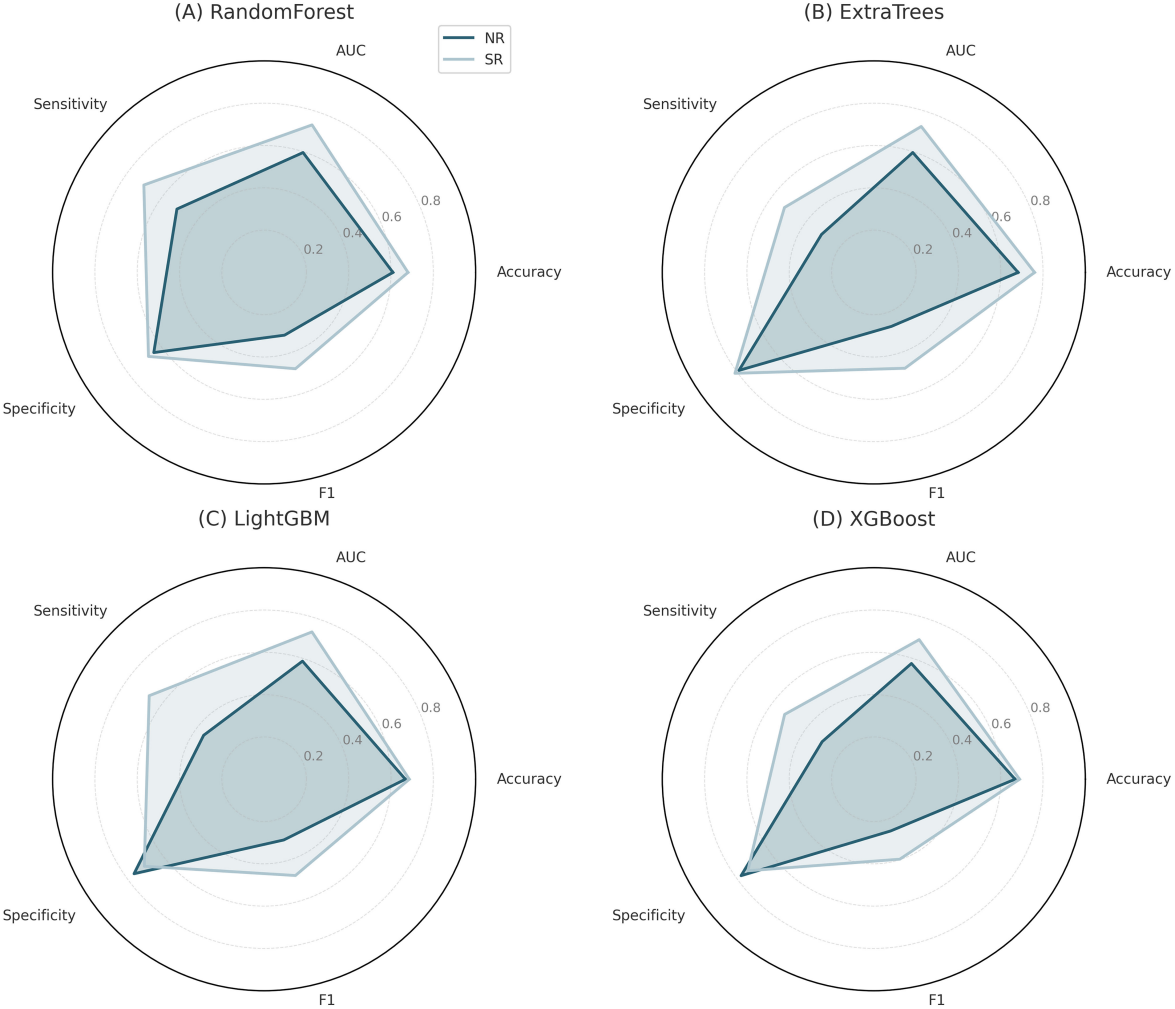
Radar charts comparing five performance metrics across four classifiers trained on super-resolution (SR) and normal-resolution (NR) ultrasound data. Each panel corresponds to a classifier: **(A)** random forest, **(B)** ExtraTrees, **(C)** LightGBM, and **(D)** XGBoost.

Overall, SR-based models outperformed NR-based models across metrics, notably AUC, sensitivity, and F1 score (**Figure 5**), underscoring the value of SR imaging for capturing fine-grained structural information that benefits radiomics-based prediction.

**Figure 6:**
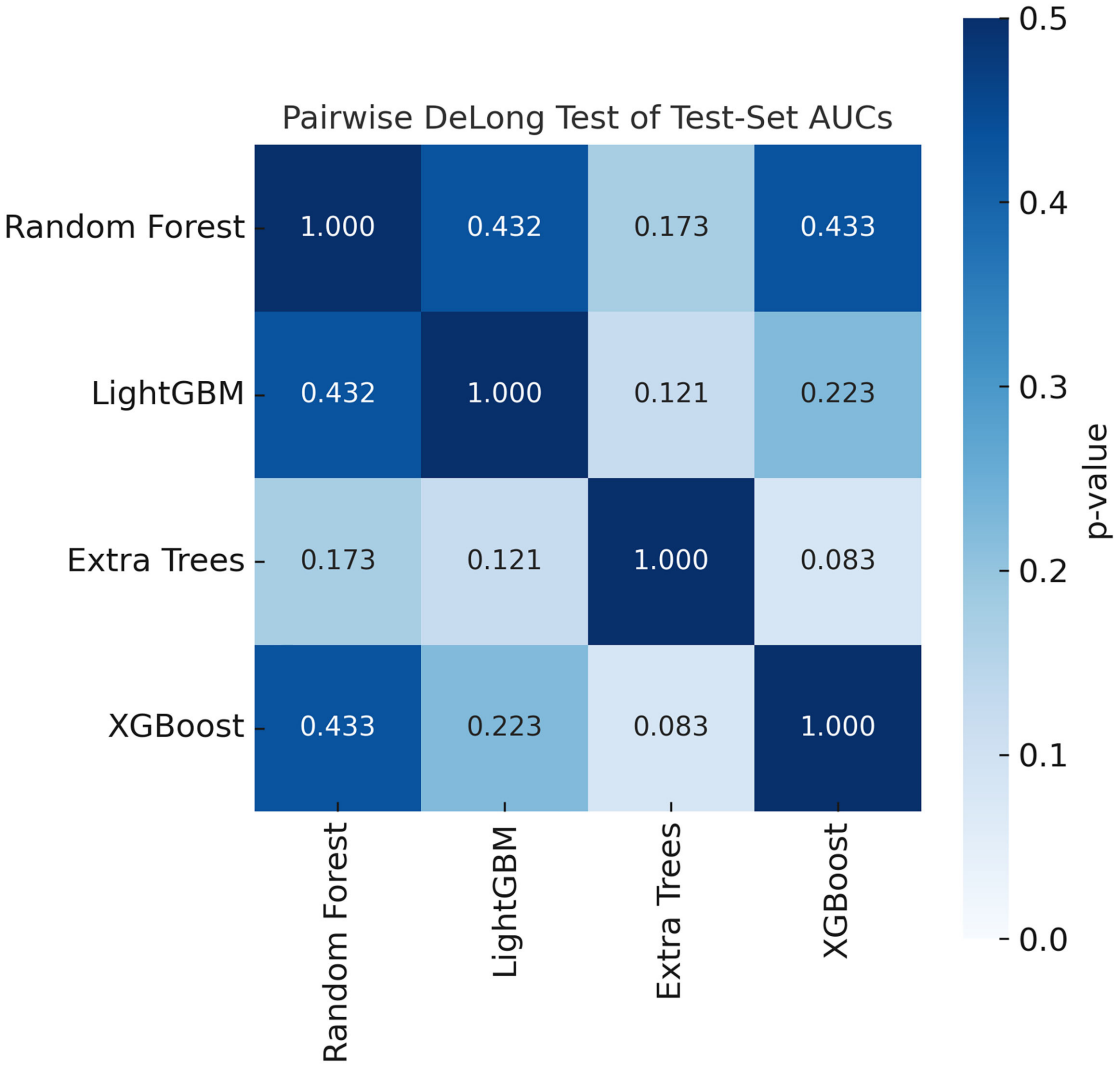
Pairwise AUC comparisons by the two-sided DeLong test among four classifiers. Cells display DeLong p values. Multiple testing was controlled by BH-FDR across the six contrasts; no comparison reached q < 0.05.

Pairwise AUC comparisons among Random Forest, LightGBM, Extra Trees, and XGBoost were evaluated using the DeLong test and summarized in **Figure 6** (cells show two-sided p values). No contrast remained significant after BH-FDR correction (raw p ≥ 0.083; q ≥ 0.335); the smallest p was observed for Extra Trees vs. XGBoost (p = 0.083).

### Net benefit evaluation

DCA was performed on the test set across threshold probabilities from 0 to 1 to evaluate clinical utility. Three clinically relevant thresholds—11%, 16%, and 21%—were selected to represent low-, moderate-, and high-intervention propensities for pre-FNA decision-making. Across this range, the RF model consistently achieved the highest net benefit, indicating an optimal balance between true-positive identification and avoidance of unnecessary procedures. All classifiers demonstrated positive net benefit across the evaluated thresholds, supporting their potential utility in clinical triage. Detailed net-benefit values with 95% CIs are provided in **Supplementary Table S9**, and the selected thresholds are indicated in **Supplementary Figure S3**.

To reflect real-world clinical class imbalance, no over- or under-sampling was applied during model training. Although an optional SMOTE step (applied post-feature selection and only within training folds) is available in the pipeline, it was disabled by default and not used in the primary analysis. This approach ensured that all reported performance metrics — including net benefit — reflect naturally imbalanced conditions and therefore provide a realistic estimate of expected model behavior in clinical deployment.

### Independent temporal validation and probability calibration

**Figure 7:**
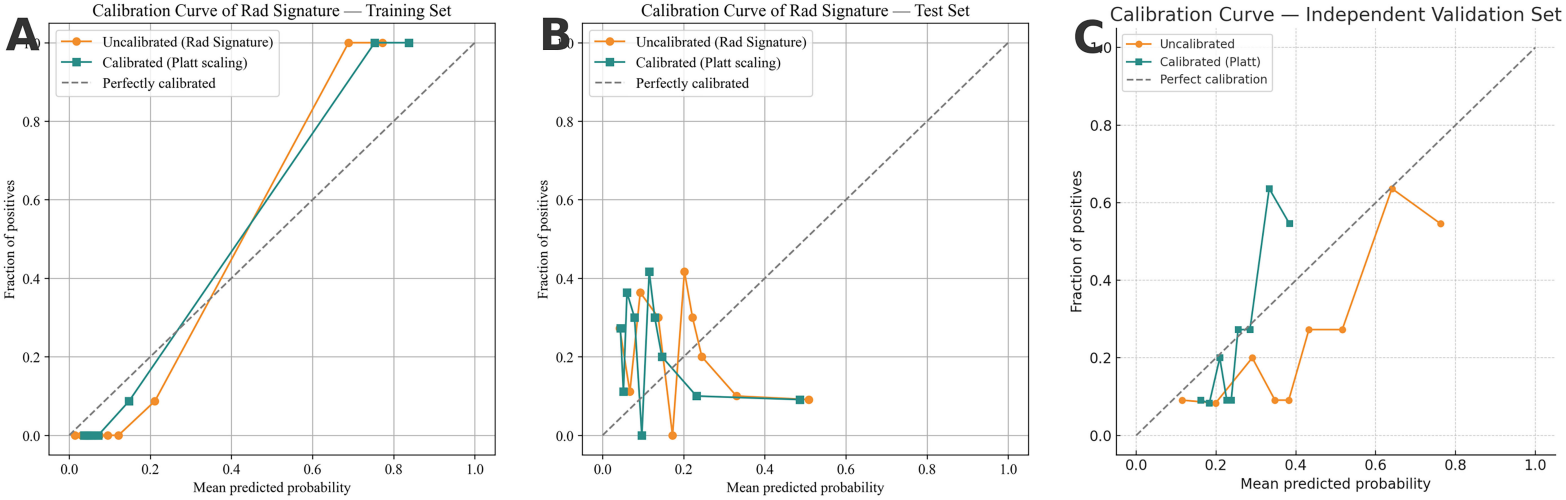
Calibration curves for the training set **(A)**, internal test set **(B)**, and independent temporal validation cohort **(C)**. Uncalibrated (orange) vs Platt-calibrated (teal); dashed diagonal = perfect calibration. Curves use 10 equal-width bins. The calibrator was fit only on the development (training/calibration) data and applied unchanged to the other sets. In **(C)**, Platt calibration reduced the Brier score from 0.1885 to 0.1702, while AUC remained 0.7435.

In the independent temporal validation cohort (n = 99; 25 Bethesda I events, 25.5% prevalence), the SR-based RF achieved an AUC of 0.7435, with the following performance: sensitivity 0.640, specificity 0.824, accuracy 0.778, PPV 0.552, NPV 0.871, and F1 0.593. At the prespecified Youden-optimal operating threshold derived from the development cohort, the corresponding confusion matrix in the temporal validation cohort was: TP = 16, FN = 9, TN = 61, and FP = 13. *Post-hoc* probability calibration using Platt scaling (sigmoid)—fit only on the development (training/calibration) set and applied unchanged to this cohort—reduced the Brier score from 0.1885 to 0.1702 while leaving AUC unchanged at 0.7435, indicating improved probability reliability without altering discrimination. Reliability diagrams (10 equal-width bins) for the training set (A), internal test set (B), and independent validation (C) cohort are shown in **Figure 7**; in each panel, the calibrated curves lie closer to the 45° reference line than the uncalibrated curves. In the temporal validation cohort, the calibration intercept was -0.85 and the calibration slope was 0.88, indicating acceptable overall calibration, with moderate global overestimation of risk and slight under-dispersion of predicted probabilities.

## Discussion

To improve pre-FNA prediction of Bethesda I outcomes in thyroid nodules, we developed and validated a SR radiomics pipeline built upon a GAN architecture. The pipeline integrated hand-crafted radiomic features extracted from both NR and SR ultrasound images using multiple machine-learning classifiers. Across all analyses, SR-based models consistently outperformed NR counterparts, aligning with previous evidence that GAN-based super-resolution enhances texture fidelity and edge definition in radiomics tasks.

A key finding was the superior discriminative ability of the SR-based models. The SR RF model achieved an AUC of 0.808 in the training set and 0.733 in the test set, whereas the NR model yielded lower values of 0.672 and 0.596, respectively. These findings are consistent with previous reports showing that GAN-based super-resolution can enhance the visibility of fine-scale textural patterns and edge sharpness in low-resolution ultrasound images (20). Improved resolution facilitates the extraction of informative features—particularly from GLRLM and GLSZM—that may be indistinct in standard-resolution images. Although NR-to-SR differences may appear subtle on visual inspection, SR emphasizes high-frequency textural components relative to conventional interpolation, thereby improving radiomic feature representation and downstream model performance.

Distinct feature compositions were observed between the NR and SR radiomics signatures. The SR signature was dominated by higher-order texture descriptors—particularly GLRLM- and GLSZM-derived metrics (e.g., original_glrlm_LowGrayLevelRunEmphasis, original_glrlm_RunLengthNonUniformity, original_glrlm_RunVariance, and original_glszm_SizeZoneNonUniformity)—together with original_firstorder_TotalEnergy. In contrast, the NR signature relied more on first-order and relatively coarse texture descriptors, including original_firstorder_Skewness, original_gldm_DependenceEntropy, original_glszm_ZonePercentage, and original_ngtdm_Busyness. Although most selected features changed in absolute magnitude after SR processing, inter-subject ordering was largely preserved (median Spearman ρ = 0.8727), indicating that SR modifies the texture representation primarily by amplifying fine-scale variations rather than by reshuffling relative differences across patients.

In addition, to qualitatively assess the behavior of the SR module and visualize where new high-frequency content is introduced, we generated pixel-wise SR–NR difference maps (**Supplementary Figure S4**). For each case, the original non–super-resolved image (NR) and the corresponding GAN-based SR reconstruction were intensity-normalized and spatially aligned. The NR frame was then upsampled 4× in-plane using bicubic interpolation to match the matrix size of the SR image, and an absolute difference map |*SR* – *NR_upsampled_*| was computed and displayed as a heat map. Across representative examples, the largest differences were consistently concentrated along tissue interfaces and at the nodule boundary, whereas the internal nodule texture and the surrounding relatively homogeneous parenchyma showed comparatively low difference values. This pattern suggests that, in our implementation, the SR module predominantly sharpens pre-existing edges and high-contrast structures rather than hallucinating entirely new intra-nodular texture patterns. These qualitative difference maps were used solely for visualization and did not enter the radiomics feature-extraction or model-training pipeline, and thus do not affect the quantitative radiomic features or model performance.

Importantly, the primary objective of this study was not to establish pixel-level physical fidelity of SR reconstructions, but rather to evaluate whether SR, as a computational image-enhancement strategy, improves downstream radiomics-based prediction of nondiagnostic cytology. In this context, SR should be interpreted as a feature-space transformation that may modify texture representation in a manner that improves predictive modeling performance, without implying a one-to-one correspondence with true histological micro-architecture. Demonstrating physical fidelity would require paired high-resolution acquisition, phantom-based validation, or controlled simulation experiments, which were beyond the scope of the present study.

Importantly, the limited feature overlap between SR (7 features) and NR (6 features), with only two shared (**Supplementary Table S5**), should not be interpreted as evidence of fundamentally different underlying biology. Radiomic texture families (GLRLM/GLSZM/GLCM/NGTDM) are highly collinear, and LASSO typically selects one representative from correlated feature clusters. Because SR reconstruction can alter local contrast, edge sharpness, and the discretized connectivity of low-contrast regions, the correlation structure among texture descriptors can shift, leading to different but related representatives being selected in SR versus NR. From a biological plausibility standpoint, SR-specific run- and zone-based metrics predominantly quantify the spatial continuity and scale heterogeneity of echogenic components. For example, original_glrlm_LowGrayLevelRunEmphasis (LGRE) emphasizes contiguous runs of low-gray-level (hypoechoic) pixels, capturing the organization of hypoechoic components rather than mean intensity alone. In thyroid ultrasound, the spatial organization of hypoechoic regions can be consistent with mixed solid–degenerative/colloid patterns and internal heterogeneity that shape acoustic scattering and grayscale texture. Nevertheless, in the absence of true high-resolution ground truth or lesion-level histopathologic correlation, these interpretations are best viewed as testable hypotheses rather than definitive claims about tissue micro-architecture.

We further compared SR-radiomics against a C-TI-RADS-type baseline logistic regression model. Using identical five independent stratified 80/20 splits, the baseline achieved a mean test AUC of 0.584 (95% CI 0.538–0.630), while the best SR-radiomics model achieved 0.733 (95% CI 0.670–0.796), supporting incremental value beyond conventional ultrasound descriptors. A potential concern is that improved performance could reflect a shortcut based on cystic composition. However, our cohort was derived from C-TI-RADS–guided FNA indications (≥4A), resulting in a predominance of solid nodules; no purely cystic nodules were included and only 18 nodules were mixed solid–cystic. Within this clinically realistic setting, variation in cystic content alone is unlikely to fully account for Bethesda I results, suggesting SR-radiomics is not merely detecting “fluid” but capturing finer heterogeneity relevant to cytological adequacy.

In the absence of lesion-level histopathologic correlation, we refrain from making causal inferences linking individual radiomic features to specific tissue substrates. Moreover, SR-induced systematic shifts in absolute feature values may affect model calibration even when discrimination remains intact; therefore, models transferred between NR and SR domains should report both discrimination and calibration, and apply *post-hoc* probability adjustment when required. Moving forward, we recommend (1) performing lesion-level histopathologic correlation in a follow-up cohort to determine which texture descriptors correspond to colloid heterogeneity, cellularity, or stromal infiltration; and (2) evaluating whether SR-based signatures enhance clinically relevant outcomes, such as reducing nondiagnostic rates, in prospective or external validation studies.

Among the evaluated classifiers, RF and LightGBM consistently outperformed ExtraTrees and XGBoost across both NR and SR datasets. RF achieved the most balanced performance, likely owing to its robustness against overfitting and ability to manage high-dimensional, partially redundant radiomic features. LightGBM also performed strongly, particularly on SR-derived features, benefiting from its leaf-wise growth strategy and efficient histogram-based splitting. In contrast, ExtraTrees and XGBoost showed lower sensitivity and generalizability, possibly because of their more aggressive splitting strategies or higher sensitivity to hyperparameter settings.

In the independent temporal validation cohort, application of the prespecified Platt calibrator—trained solely on the training/calibration set and applied unchanged—reduced the Brier score from 0.1885 to 0.1702, while the AUC remained 0.7435, indicating improved probability reliability without compromising discrimination. Reliability diagrams (10 equal-width bins) for the training, test, and independent cohorts demonstrated calibrated curves consistently closer to the 45° reference line, as expected for sigmoid calibration, which remaps predicted probabilities while largely preserving rank order. Minor residual deviations likely reflected temporal shifts in case mix or prevalence and finite-sample variability, particularly within extreme bins. Practically, we recommend using calibrated probabilities for threshold selection and individualized risk communication, deploying the model together with the fitted Platt mapping, and instituting routine calibration monitoring (e.g., Brier scores and reliability plots) with periodic recalibration when drift is detected.

**Figure 8:**
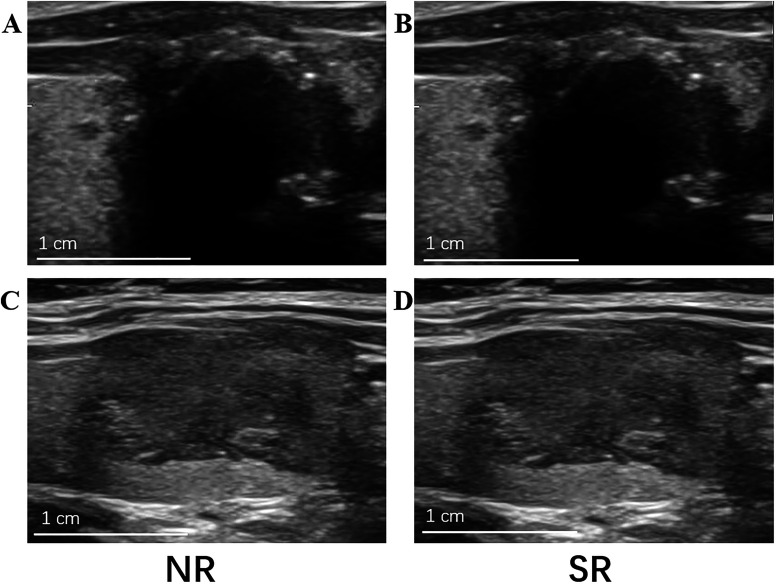
Misclassified cases in Bethesda I prediction: NR vs SR. Paired NR (left) and SR (right) images are shown for representative misclassified examples. The upper row shows a false-positive (FP) case and the lower row shows a false-negative (FN) case. **(A, B)** are the FP case in NR and SR, respectively; **(C, D)** are the FN case in NR and SR, respectively. For each case, the field-of-view and display settings were kept identical. Scale bar: 1 cm.

A qualitative review of misclassified cases offers insight into model behavior. Among false positives—predicted as nondiagnostic (Bethesda I) but ultimately diagnostic—we frequently observed nodules with prominent cystic components, marked heterogeneity, or peripheral rim calcification. The dense echogenic ring and associated posterior acoustic shadowing in rim-calcified nodules can obscure internal parenchymal texture, generating homogeneous or low-contrast regions that the model may misinterpret as indicative of low cellularity or inadequate sampling potential. Similarly, pronounced posterior acoustic effects—such as shadowing from coarse calcifications, posterior enhancement, or reverberation and comet-tail artifacts—can introduce artificial texture regularities that act as spurious risk cues. In contrast, false negatives—predicted as diagnostic but ultimately nondiagnostic—were predominantly isoechoic or mildly hypoechoic nodules with homogeneous internal texture and indistinct margins. The absence of pronounced structural gradients or internal heterogeneity led to feature profiles resembling those of confidently diagnostic cases, causing the model to underestimate nondiagnostic risk (**Figure 8**). Moreover, suboptimal delineation of ill-defined boundaries and partial-volume effects from adjacent parenchyma could further blur radiomic contrast, attenuating the discriminative power of second-order texture descriptors even after SR enhancement.

Importantly, within our cohort, we did not observe a systematic operator effect on model errors during ultrasound acquisition; error patterns were comparable across operators using standard presets. By contrast, cytological adequacy is influenced by factors beyond imaging—such as sampling technique, targeting strategy and number of needle passes, specimen preparation/handling, and the presence or absence of on-site adequacy assessment—which were not explicitly modeled and may partly account for nondiagnostic outcomes independent of imaging characteristics. We also conducted an exploratory analysis of operator experience quantified by the EWI and did not find a statistically significant association with Bethesda I outcomes, nor did EWI differ significantly between the Bethesda I and non-I groups. This null finding should be interpreted cautiously because operator experience in a single-center setting may be relatively homogeneous (limiting analyzable variance), EWI is a relatively coarse surrogate that may not capture key process-level determinants of adequacy, and the number of Bethesda I events may have limited statistical power to detect small-to-moderate effects. Accordingly, we consider the EWI results exploratory and plan future prospective studies incorporating more granular procedural variables to more comprehensively evaluate operator-related contributions to sampling adequacy.

Recent multicenter studies underscore that conventional (non-GAN) ultrasound radiomics and broader artificial intelligence and machine learning (AI/ML) frameworks can generalize effectively across institutions. For instance, a three-center Scientific Reports study developed a PyRadiomics-based model to predict preoperative extrathyroidal extension in papillary thyroid carcinoma, achieving AUCs of 0.841 (internal test) and 0.814 (external validation) using LASSO/principal component analysis with XGBoost, thereby demonstrating the cross-site robustness of conventional radiomics pipelines (21). In benign–malignant classification, a three-hospital study combining intranodular and perinodular ultrasound radiomics achieved a test-set AUC of 0.933 when integrated with clinical variables, notably without any SR preprocessing (22). Multicenter, multimodal ultrasound radiomics—combining grayscale and shear-wave elastography—has likewise demonstrated strong performance in TI-RADS 4–5 nodules (23). Beyond radiomics, large-scale multicenter AI studies—including a national retrospective–prospective trial linking deep learning with FNA cytology and a five-hospital model for subcentimeter nodules—further demonstrate external validity at scale (24). Against this backdrop, our SR radiomics approach addresses a distinct clinical question—pre-FNA prediction of nondiagnostic (Bethesda I) outcomes—yet the observed superiority of SR over NR models suggests that SR can be integrated into established non-GAN radiomics or AI pipelines. Multicenter validation across institutions and scanners remains necessary to confirm that these performance gains are robust to domain shift.

For nodules identified by the model as high risk for nondiagnostic (Bethesda I) sampling, model outputs may be translated into actionable procedural strategies within a standardized clinical workflow. At a prespecified probability threshold (e.g., the Youden-optimal cut-off derived from the development cohort), nodules exceeding this risk level could be triaged to intensified sampling protocols, including the implementation of rapid on-site evaluation (ROSE), additional needle passes during the same session, adjustment of needle gauge or aspiration technique, or early repeat FNA within a predefined timeframe (e.g., 2–6 weeks). In higher-risk scenarios or in cases with repeated nondiagnostic results, escalation to ultrasound-guided core-needle biopsy or adjunctive molecular testing may be considered according to institutional practice.

Operationally, integration into electronic health record systems could enable automatic flagging of high-risk nodules, expedited scheduling, and predefined escalation criteria, thereby standardizing decision-making and reducing procedural variability. Based on the observed prevalence of Bethesda I nodules and the model’s validation sensitivity and specificity, such deployment may facilitate earlier identification of patients at risk for nondiagnostic sampling and potentially reduce repeat procedures or diagnostic delays. However, the magnitude of clinical impact would depend on local workflow patterns and should be prospectively evaluated. Importantly, the model is intended as an adjunctive risk stratification tool rather than a replacement for clinical judgment or TI-RADS–based assessment.

This study has several limitations that should be acknowledged. First, this was a single-center study conducted on a single ultrasound platform (GE LOGIQ E9), and therefore the findings primarily reflect the imaging characteristics and acquisition presets of this specific vendor environment. Ultrasound image formation is highly dependent on device-specific signal processing pipelines, beamforming algorithms, post-processing filters, and grayscale mapping strategies. These factors can alter intensity distributions, textural statistics, and gray-level discretization behavior, potentially leading to domain shift when models are transferred across scanners or institutions. This concern may be particularly relevant for GAN-based super-resolution. Although SR was applied in inference-only mode, the pretrained generator implicitly encodes texture priors learned from its original training distribution. When applied to ultrasound images acquired from different vendors or with different preset configurations, SR reconstruction may amplify vendor-specific high-frequency patterns or noise characteristics, thereby modifying radiomic texture descriptors in a device-dependent manner. Consequently, the performance gains observed in this study should not be assumed to directly generalize across ultrasound systems without empirical external validation. Accordingly, the present model should be interpreted as platform-specific at this stage, and external validation across multiple institutions and ultrasound systems is necessary before clinical deployment.

Second, Bethesda I nodules did not undergo surgical resection; therefore, lesion-level histopathologic confirmation was unavailable. Nondiagnostic status was defined cytologically, precluding direct pathological correlation with radiomic features and necessitating cautious interpretation of biological relevance. In particular, the absence of paired high-resolution ultrasound or histologic ground truth prevents definitive validation that SR-enhanced high-frequency textures correspond to true tissue micro-architecture rather than model-driven representations. Although qualitative SR–NR difference maps suggested that the most prominent changes occurred along tissue interfaces and nodule boundaries, subtle GAN-induced high-frequency components may still influence certain texture descriptors in ways that are not visually apparent. Accordingly, SR in this study should be regarded as an image enhancement strategy for radiomic modeling rather than a pixel-level surrogate for histologic structures.

Third, although an AUC of 0.73–0.74 is acceptable, the modest decrease in performance from the training to the internal test sets suggests a degree of performance optimism, which is expected given the limited sample size (n = 338) and positive cases (n = 73). While repeated stratified 80/20 splitting reduces information leakage by strictly separating training and test partitions, this resampling strategy does not fully eliminate model-selection optimism introduced by the multi-stage feature selection pipeline (Mann–Whitney U filtering, correlation pruning, RFE, and LASSO regularization). Consequently, the reported AUC values represent internally validated performance estimates based on repeated stratified resampling and independent temporal validation. To enhance transparency, we quantified performance variability across five independent resampling iterations and observed a mean test AUC of 0.728 ± 0.062. Furthermore, we included an independent temporal validation cohort (n = 99), providing an out-of-time assessment of generalizability. Future studies with larger multicenter datasets may incorporate additional validation strategies (e.g., nested cross-validation or bootstrap-based approaches) to further refine generalization estimates.

Fourth, cytological adequacy is influenced by non-imaging factors—such as operator technique, targeting strategy, number of needle passes, and specimen handling—that were not explicitly modeled in this study. Regarding SR, we employed a fixed, pre-specified implementation without parameter modification or dataset-specific fine-tuning. Although this approach enhances procedural transparency and reproducibility, it may leave domain misalignment unresolved across devices or populations. Future work should incorporate (i) phantom and multi-vendor evaluations, (ii) limited device-aware calibration or fine-tuning of the SR module when necessary, (iii) harmonized intensity normalization and gray-level discretization across scanners, and (iv) domain adaptation strategies to minimize inter-site variability.

## Conclusion

SR radiomics significantly enhances the pre-FNA prediction of Bethesda I outcomes in thyroid nodules compared with conventional normal-resolution analysis. This approach may help reduce nondiagnostic procedures and facilitate more informed clinical decision-making. Further evaluation in larger, multicenter studies is warranted.

## Data Availability

The dataset contains sensitive clinical and imaging information from human subjects and is not publicly available due to institutional privacy regulations and ethical restrictions. Access to the dataset may be granted upon reasonable request to the corresponding author and with approval from the appropriate ethics committee. Requests to access the dataset should be directed to: JS, Email: shengjunfa1026@163.com.

## Supplementary material

Supplementary Figure S1 Heatmap of radiomic feature reproducibility (ICC). Heatmap of intra- and inter-observer intra-class correlation coefficients (ICCs) for all 107 radiomic features. Features with ICC ≥ 0.75 in both assessments (n = 82) were considered reproducible and retained for subsequent modeling.

Supplementary Figure S2 Paired SR–NR comparisons for LASSO-selected radiomic features. Paired SR–NR comparisons and Bland–Altman plots for the eleven radiomic features in the final LASSO-derived signatures, showing systematic SR–NR shifts in absolute values while preserving inter-subject ranking (as reflected by Spearman correlation coefficients in **Supplementary Table S6**).

Supplementary Figure S3 Decision curve analysis at clinically relevant thresholds. Decision curve analysis (DCA) for the machine-learning classifiers on the test set across threshold probabilities from 0 to 1, including treat-all and treat-none strategies. Three clinically relevant thresholds (11%, 16%, and 21%) for pre-FNA decision-making are highlighted, and the random forest (RF) model shows the highest net benefit in this range. Net-benefit values with 95% confidence intervals are summarized in **Supplementary Table S9**.

Supplementary Figure S4 Qualitative SR–NR difference map for a representative thyroid nodule**. (A)** Original non–super-resolved (NR) ultrasound image. **(B)** Corresponding GAN-based super-resolution (SR) reconstruction. **(C)** Absolute pixel-wise difference map |SR − NR_upsampled|. The NR frame was upsampled 4× in-plane using bicubic interpolation to match the SR matrix size, and the absolute difference was displayed as a heat map, with larger differences mainly along tissue interfaces and the nodule boundary.
