# Supplementary figures and images for: Super-resolution ultrasound radiomics for pre-FNA prediction of nondiagnostic (Bethesda I) thyroid nodules
Source: Front Endocrinol (Lausanne). 2026 May 1;17:1710097. doi: 10.3389/fendo.2026.1710097 (PMC13175800; doi:10.3389/fendo.2026.1710097)

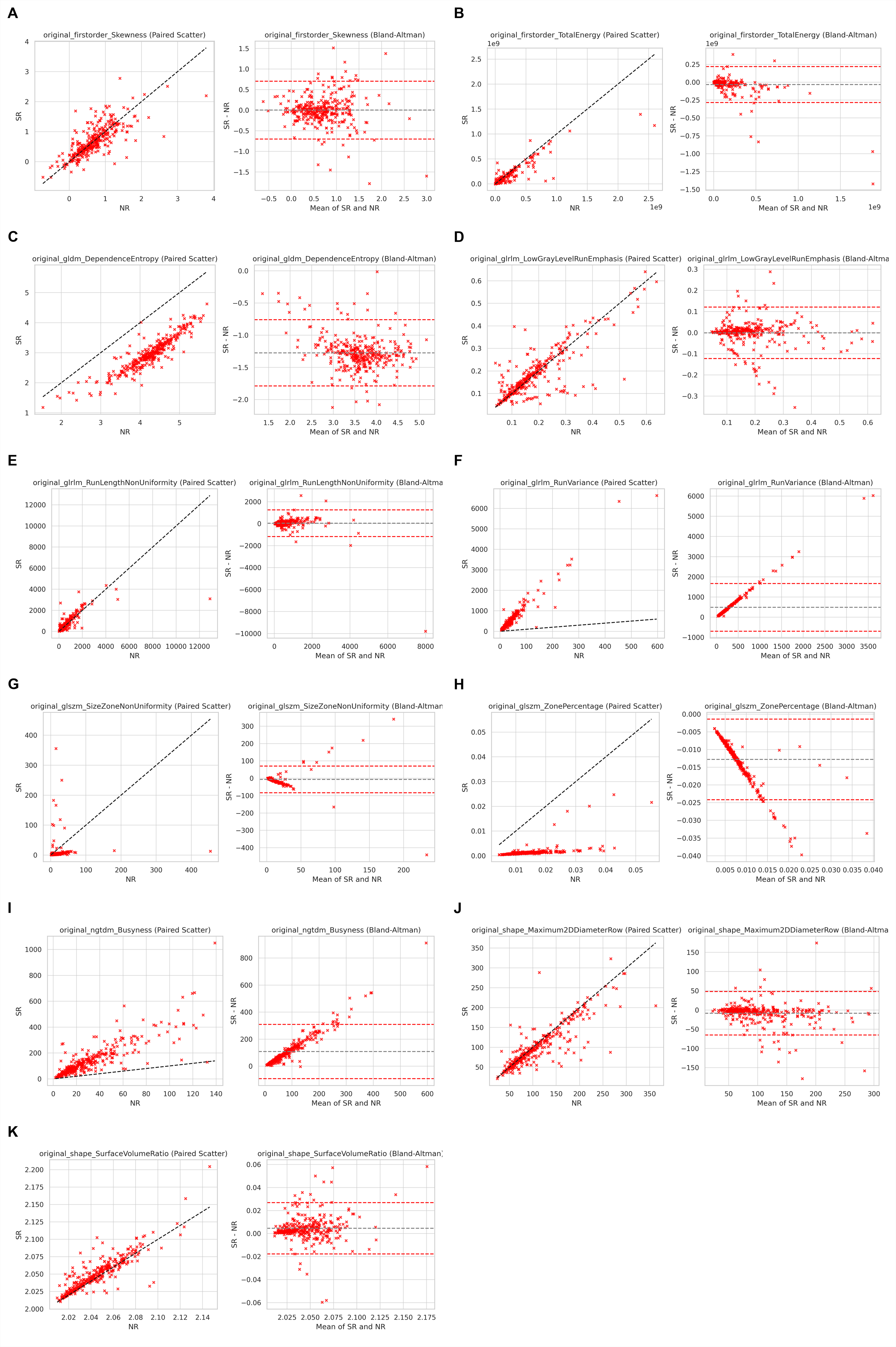

Supplement: Supplementary Figure S2 — Paired SR–NR comparisons for LASSO-selected radiomic features. Paired SR–NR comparisons and Bland–Altman plots for the eleven radiomic features in the final LASSO-derived signatures, showing systematic SR–NR shifts in absolute values while preserving inter-subject ranking (as reflected by Spearman correlation coefficients in Supplementary Table S6). [file Image2.tif]

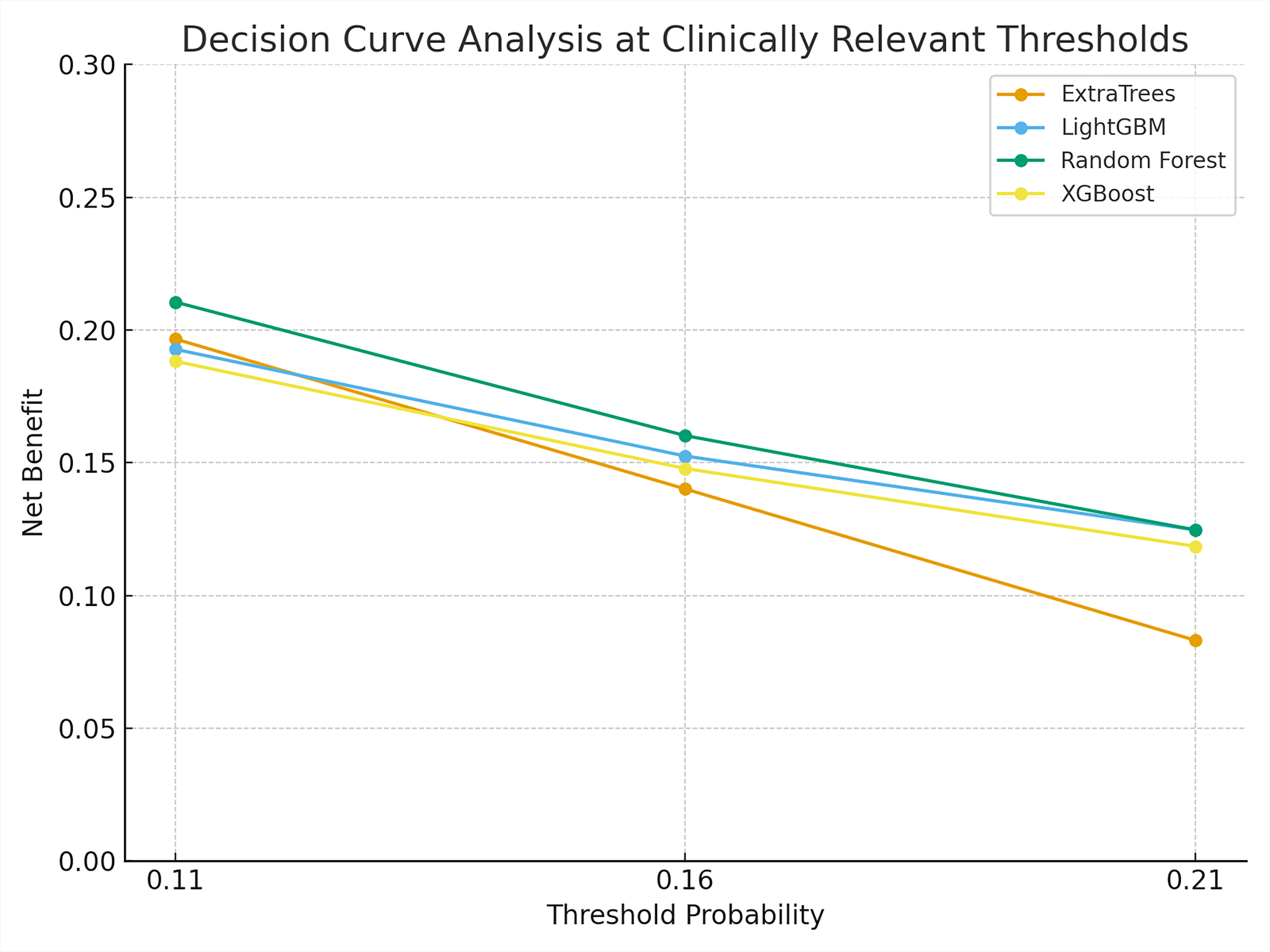

Supplement: Supplementary Figure S3 — Decision curve analysis at clinically relevant thresholds. Decision curve analysis (DCA) for the machine-learning classifiers on the test set across threshold probabilities from 0 to 1, including treat-all and treat-none strategies. Three clinically relevant thresholds (11%, 16%, and 21%) for pre-FNA decision-making are highlighted, and the random forest (RF) model shows the highest net benefit in this range. Net-benefit values with 95% confidence intervals are summarized in Supplementary Table S9. [file Image3.tif]

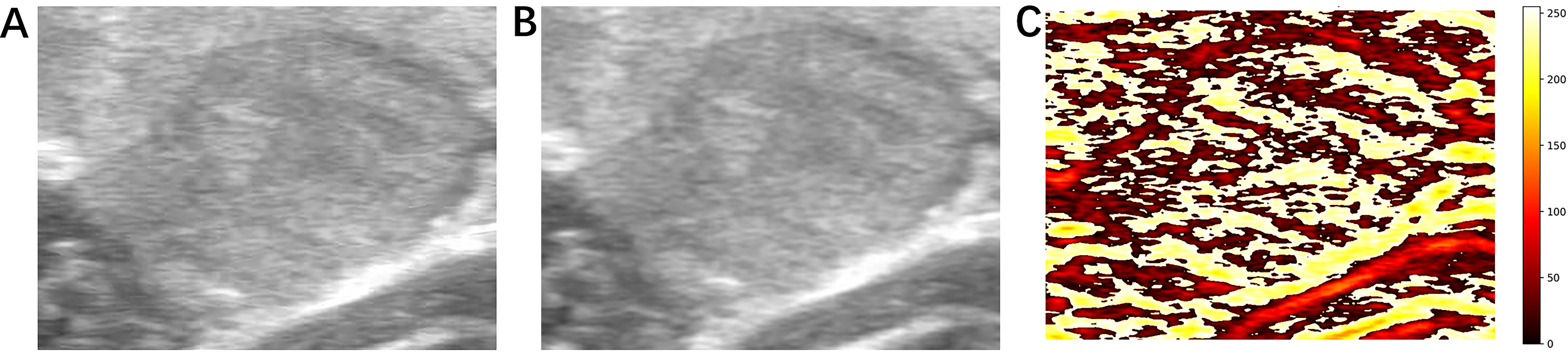

Supplement: Supplementary Figure S4 — Qualitative SR–NR difference map for a representative thyroid nodule. (A) Original non–super-resolved (NR) ultrasound image. (B) Corresponding GAN-based super-resolution (SR) reconstruction. (C) Absolute pixel-wise difference map |SR − NR_upsampled|. The NR frame was upsampled 4× in-plane using bicubic interpolation to match the SR matrix size, and the absolute difference was displayed as a heat map, with larger differences mainly along tissue interfaces and the nodule boundary. [file Image4.tif]
